# Medium Preparation for the Cultivation of Microorganisms under Strictly Anaerobic/Anoxic Conditions

**DOI:** 10.3791/60155

**Published:** 2019-08-15

**Authors:** Andreas O. Wagner, Rudolf Markt, Mira Mutschlechner, Nina Lackner, Eva M. Prem, Nadine Praeg, Paul Illmer

**Affiliations:** 1 Department of Microbiology, Universität Innsbruck

**Keywords:** Biochemistry, Issue 150, anaerobic, anaerobic cultivation, anaerobic digestion, biogas, media preparation, resazurin, batch, gas, liquid sampling

## Abstract

In contrast to aerobic organisms, strictly anaerobic microorganisms require the absence of oxygen and usually a low redox potential to initiate growth. As oxygen is ubiquitous in air, retaining O_2_-free conditions during all steps of cultivation is challenging but a prerequisite for anaerobic culturing. The protocol presented here demonstrates the successful cultivation of an anaerobic mixed culture derived from a biogas plant using a simple and inexpensive method. A precise description of the entire anoxic culturing process is given including media preparation, filling of cultivation flasks, supplementation with redox indicator and reducing agents to provide low redox potentials as well as exchanging the headspace to keep media free from oxygen. Furthermore, a detailed overview of aseptically inoculating gas tight serum flasks (by using sterile syringes and needles) and suitable incubation conditions is provided. The present protocol further deals with gas and liquid sampling for subsequent analyses regarding gas composition and volatile fatty acid concentrations using gas chromatography (GC) and high performance liquid chromatography (HPLC), respectively, and the calculation of biogas and methane yield considering the ideal gas law.

## Introduction

On earth molecular oxygen in notable concentrations is available in areas having direct contact with the atmosphere or in the presence of oxygenic phototrophs. Environments in which oxygen is absent are called anaerobic. However, energy conversion is still possible under anaerobic conditions via two different metabolic processes, fermentation and anaerobic respiration^1^.

While organisms undergoing aerobic respiration are using oxygen as a terminal electron acceptor, anaerobic respiration requires alternative electron acceptors like nitrate or sulphate^2^. In the so-called “electron tower”, redox couples are organized according to their redox potential, with the most negative ones located at the top (electron donors) and strongest oxidation agents with positive redox potential at the bottom (electron acceptors). The electron transfer between donors and acceptors leads to energy conservation via the so-called respiratory chain and electrons can be captured by electron acceptors - to stay in the picture - in different floors of the tower. Thereby, the higher the fall of electrons through the electron tower, the more energy can be conserved by the respective reaction. Therefore, respiration is also possible in anaerobic habitats, for example, with redox pairs including NO_3_^-^/NO_2_^-^, fumaric acid/succinic acid, SO_3_^2-^/H_2_S, S°/H_2_S, Mn(IV)/Mn(II), Fe(III)/Fe(II)^2,3^. First, the resulting energy is conserved as membrane potential, which is subsequently used by electron transport phosphorylation for adenosine-triphosphate (ATP) synthesis by membrane-bound ATP-synthases. In contrast to aerobic respiration, the amount of energy that can be conserved by anaerobic respiration can be dramatically reduced; however, the energy output of most anaerobic respirations is still higher compared to fermentation, an anaerobic energy conservation path in habitats lacking oxygen and other terminal electron acceptors^2^.

During fermentation, energy-rich, organic substrates are degraded to various fermentation products that often define the name of the overall process, for example, alcoholic fermentation. In contrast to respiration processes, ATP generation during fermentation is limited to substrate-level phosphorylation during which a phosphate group is transferred to adenosine-di-phosphate (ADP) from an energy-rich phosphorylated substrate^2^. Fermenting microorganisms play a central role in the anaerobic degradation of organic matter as they are key-players in substrate breakdown. The primary fermentation products, like organic acids, alcohols, CO_2_, and H_2_, can subsequently be used by secondary fermenting microorganisms to produce acetic acid, CO_2_, and H_2_. Examples for fermentation products include lactic acid, various volatile fatty acids (formic-, acetic-, propionic-, butyric-, valeric acid), n-butanol, 2,3-butandiol, acetone, and ethanol.

Cultivation of microorganisms under strictly anaerobic conditions requires completely different methods and equipment compared with the cultivation of aerobic organisms. While oxygen-tolerant organisms are often cultivated on agar dishes, so-called surface cultures, this is - with a few exceptions - hardly possible for strictly anaerobic microorganisms. Therefore, enrichment cultures of strictly anaerobic microorganisms are mainly established in liquid media applying culture vessels sealed with gas-tight septa that ensure an oxygen-free headspace atmosphere^4,6,7^.

The current protocol description will provide appropriate cultivation methods for target microorganisms of a mixed population derived from an anaerobic biogas plant. The isolation and cultivation of pure cultures is even more challenging but not part of this work.

Here, we show the procedure for cultivating an anaerobic microbial community based on a study regarding the formation of phenyl acids during anaerobic digestion of proteinaceous substrates^8^. The microbial community consisted of members from all four phases of anaerobic digestion: hydrolysis, acidogenesis, acetogenesis, and methanogenesis. A mineral salt medium supplemented with a carbon source, redox-indicator, vitamin and trace element solution, and reducing agent was applied^9^. The medium was amended with the respective proteinaceous phenyl acid precursor substrates^8^.

## Protocol

### 1 Preparation of medium

1. Prepare redox indicator stock solution (0.1 g of resazurin/100 mL aqueous solution).
2. Prepare vitamin solution (Table 1).
3. Prepare trace element solution (Table 2).
  NOTE: The order of addition is important; please refer to Table 2 and respective protocols.
4. Prepare reducing agent stock solution (60 g Na2S/L aqueous solution).
5. Weigh medium ingredients (mineral salt medium, Table 3) in an appropriate flask (e.g., 1 L screw cap lab flask).
  NOTE: Depending on the experimental setup, the addition of a separate carbon source might be necessary.
6. Add half-volume of distilled water (Table 3) and dissolve the ingredients.
7. Add 1 mL of redox indicator solution according to Table 3.
8. Add vitamin and trace element solution according to Table 3.
9. Adjust the pH according to medium/organism requirements in Table 3.
  NOTE: Color of redox indicator is pH dependent and might require some time to adjust.
10. Bring to a final volume of 1 L with distilled water.
  NOTE: Vitamin and trace element solutions can also be added after autoclaving by aseptically adding a filter-sterilized aliquot (diluted solutions, filter pore size < 0.2 µm) into previously closed and autoclaved serum flasks. However, this approach bears an elevated risk of contamination.

### 2 Filling of cultivation flasks

1. Thoroughly clean and dry 120 mL serum flasks.
  NOTE: Serum flasks are available in different volume capacities (e.g., 20, 60, 120, 250 mL).
2. Thoroughly clean and dry butyl rubber septa.
3. Weigh additional medium components (e.g., phenyl acid precursor substrates) in the cultivation flasks.
  NOTE: Additional components depend on experimental setup and hypothesis.
4. Fill the serum flasks with 50 mL of medium.

### 3 Reduction/removal of oxygen in the liquid phase

1. Prepare a ~100°C water bath.
2. Set filled serum flasks in the water bath and incubate for approx. 20-30 min to reduce the solubility of O_2_ in the liquid phase.
3. Flush the headspace immediately with N_2_ gas or alternatively with other gas or gas mixtures like N_2_/CO_2_.
  CAUTION: Take care of appropriate room ventilation.
4. Close the flasks with butyl rubber septa and fix with aluminum caps.
  NOTE: Rubber septa might often fit better onto the neck of the flask by adding a drop of water/medium while drilling it in.
5. Add 0.1 mL of reducing agent (stock solution) to each flask filled with 50 mL of medium to further reduce the redox potential (0.1 mL of reducing agent per 50 mL of medium).
6. Autoclave for 20 min at 121°C.
  CAUTION: An autoclave certified for the sterilization of closed vessels has to be used. Otherwise, overpressure derived from temperature increase might cause serum flasks to explode.

### 4 Inoculation of the medium

1. Prepare inoculum from anaerobic digester.
  1. Add 400 mL of distilled water into a flask and bring it to boil.
  2. Cool it down (< 30°C) while permanently flushing the headspace with N_2_.
  3. Add approx. 100 g of sludge derived from an anaerobic digester.
    NOTE: Avoid excessive contact of sludge with oxygen.
  4. Record the exact mass of added sludge for the exact determination of dilution.
  5. Exchange the flask’s headspace with N_2_ and close it with a butyl rubber septum.
  6. Shake the flask for 30 min at 120 rpm.
2. Remove 5 mL inoculum by using syringe + cannula and inject it into prepared serum flasks as described in step 1-3.

### 5 Incubation, sampling, and analysis

1. Incubate inoculated serum flasks at a temperature that is appropriate for the respective experiment.
  NOTE: Incubation temperature is dependent on experimental setup and used inoculum.
  1. Drain overpressure resulting from the temperature increase using syringe + cannula, when the liquid in the serum flasks has equilibrated to incubation temperature (about 15 – 30 min, depending on incubation temperature).
    CAUTION: Depending on the applied substrate, its concentration, temperature, incubation time, inoculum type and concentration, overpressure within flasks can rise by up to > 2 bar pressure and might cause serum flaks to explode. Monitoring the overpressure by using a manometer and subsequently draining overpressure with a cannula is therefore mandatory.
2. Evaluate biogas production and composition during incubation time.
  NOTE: Incubation period can span a few days to several weeks.
  1. Record current atmospheric pressure.
  2. Prepare a manometer and evaluate the pressure within the flasks derived from microbial activity.
  3. Shake the flasks.
  4. Remove 1 mL of headspace gas using a syringe + cannula, and measure the H_2_, O_2_, CH_4_, and/or CO_2_ concentrations via gas chromatography.
    NOTE: For the qualification and quantification of H_2_, O_2_, CH_4_, and CO_2_, a gas chromatograph was used applying operation temperatures of 160°C (column oven), 100°C (injector), and 180°C (thermal conductivity detector, TCD). N_2_ was used as a carrier gas. For details, refer to previous studies^10^.
3. Monitor concentrations of volatile fatty acids (VFA) and phenyl acids. For VFA and phenyl acid analysis, use a HPLC system equipped with an UV-detector (at 220 nm) running with 5 mM H_2_SO_4_ as a mobile phase. For method’s details and additional information on sample storage, please refer to previous studies^11^.
  NOTE: The analysis of VFA is exemplary for many other physico-chemical analyses or microscopic evaluations. Moreover, molecular biological methods targeting the abundance of specific microorganisms and/or composition of the microbial community at a certain point of the experiment can be applied using the described procedure.
  1. Remove 1 mL of liquid with syringe + cannula.
    NOTE: Samples can be frozen (-20°C) immediately after withdrawal and analyzed at the end of the experiment^11^.
  2. Centrifuge at 15,000–20,000 x *g* and pass through 0.2 µm RC (regenerated cellulose) filters.
  3. Inject 5-20 µL onto a HPLC system and analyze for VFA composition and concentration of phenyl acids.
4. Drain flask’s overpressure using a cannula.
  NOTE: After determining the pressure and gas composition as well as taking any necessary sample, place the cultivation flask back on respective temperature and do not drain overpressure before the liquid has achieved the incubation temperature.
5. Calculate biogas and methane production V_CH4N_ considering the ideal gas law using Equation 1-3. Please also refer to Table 4.
  Equation 1: ${\text{V}}_{\text{CH}4\text{N}}={\text{V}}_{\text{CH}4\text{T}}-{\text{V}}_{\text{CH}4\text{R}}$
  whereby
  Equation 2: ${\text{V}}_{\text{CH}4\text{T}}=\frac{\left(\left({\text{p}}_{\text{M}}+{\text{p}}_{\text{S}}+{\text{p}}_{\text{A}}-{\text{p}}_{\text{AX}}\right)*{\text{V}}_{\text{H}}\right)*{\text{T}}_{\text{S}}}{{\text{T}}_{I}*{\text{p}}_{\text{S}}}*\text{C}{\text{H}}_{4\text{\%}}/100$
  and
  Equation 3: $V_{\text{CH}4R}=\frac{p_{AX}*V_{HX}*T_{S}}{T_{I}*p_{S}}*CH_{4\%X}/100$
  NOTE: For the calculation of the total biogas production, the amount of CH_4%_ and CH_4%X_ in equation 2 and 3 has to be set to 100. NmL: normalized gas volume under standardized conditions (0°C, 1 atm), under which the molar gas volume is 22.414 NmL/mmol.

## Representative Results

**Figure 1:**
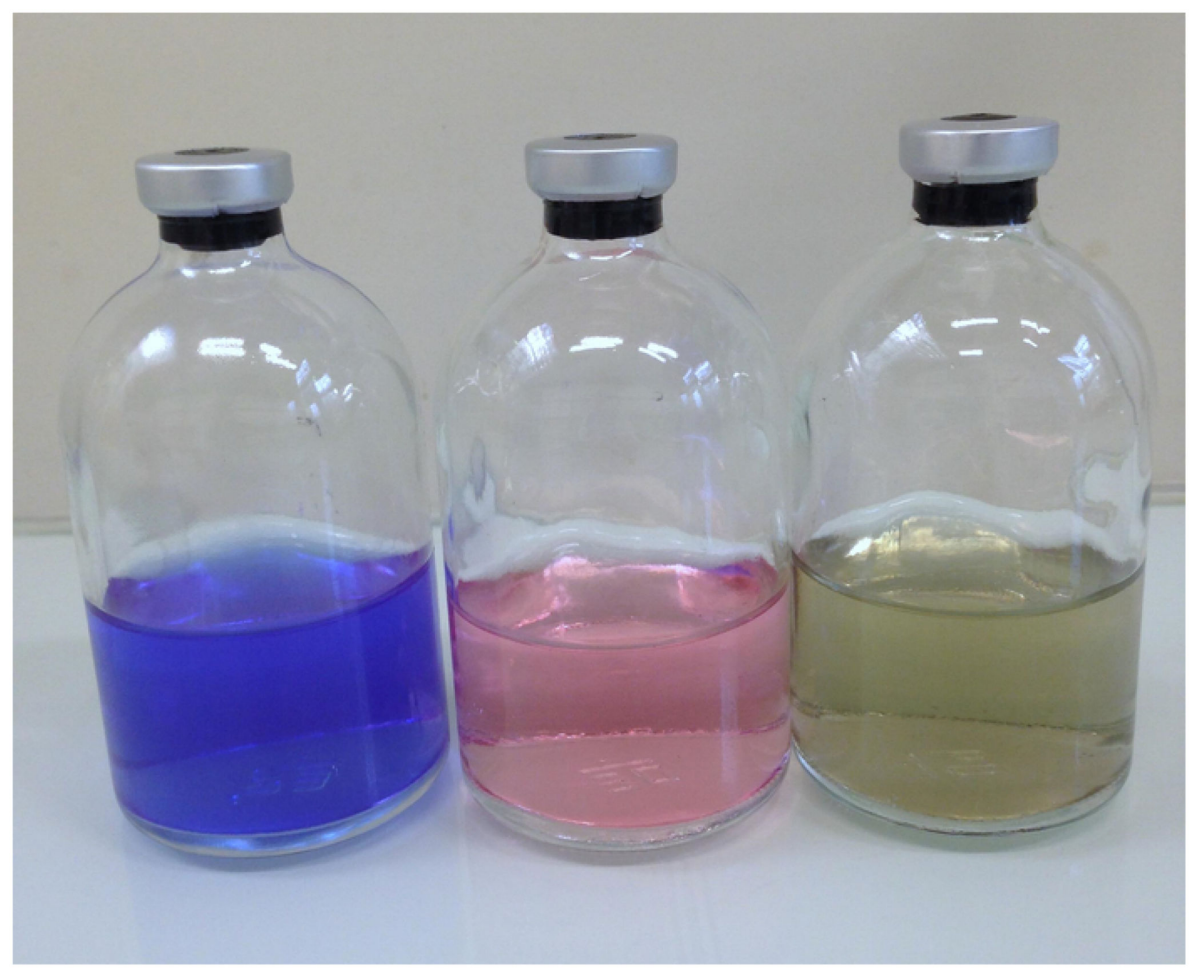
Redox indicator. Correct redox potential in the cultivation flasks can be controlled by adding a redox indicator. Please click here to view a larger version of this figure.

**Figure 2:**
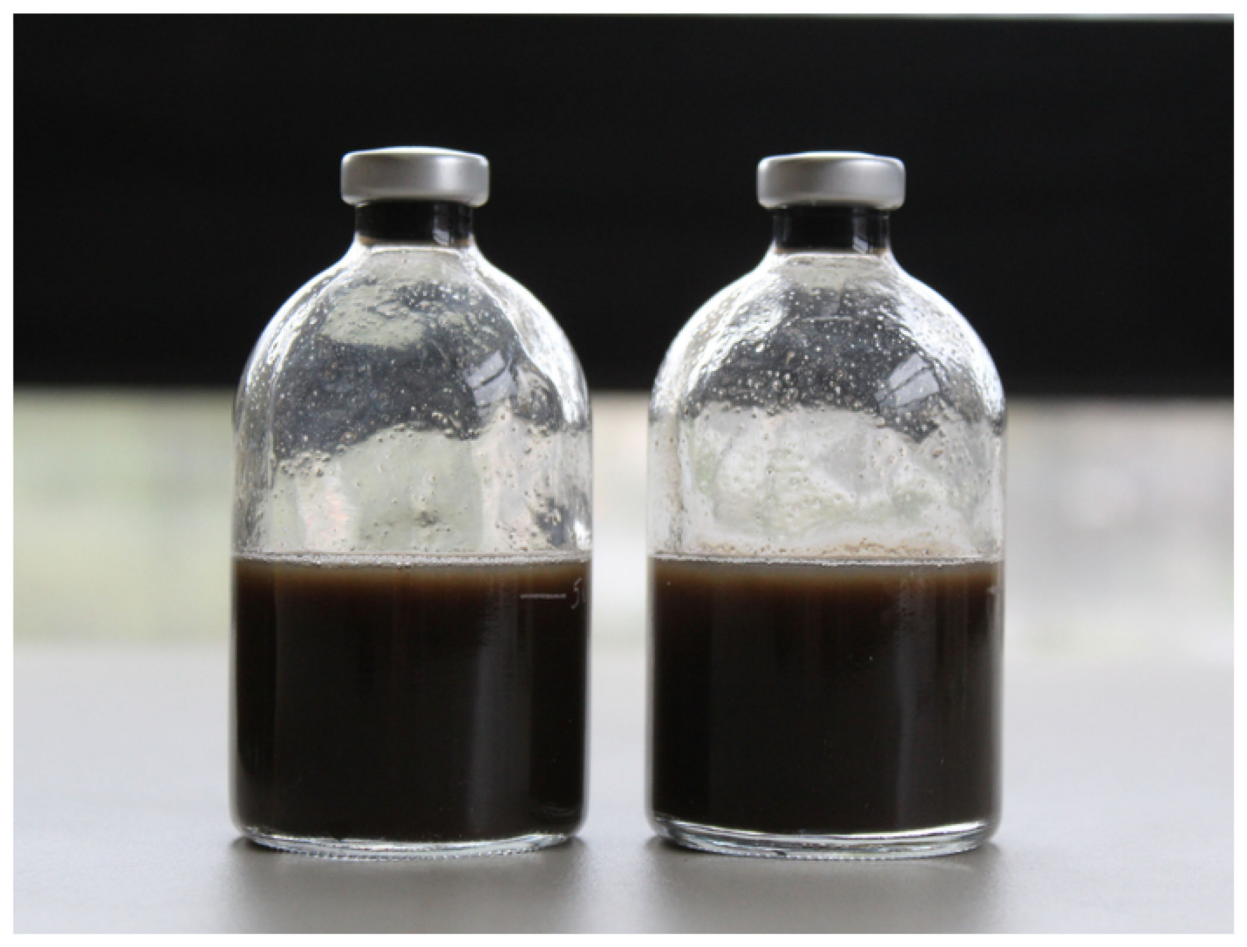
Miniature batch bioreactors. Miniature batch bioreactors prepared in 120 mL cultivation flasks for anaerobic digestion experiments. Flasks were filled with medium and inoculated with diluted digester sludge. The reactors were gas-tightly sealed with butyl rubber stoppers and aluminum caps. Please click here to view a larger version of this figure.

Cultivation flasks were filled with medium under anaerobic conditions according to the protocol described above, checked for the appropriate color (Figure 1), and used as miniature batch bioreactors conducting anaerobic digestion. These were amended with substrates potentially causing phenyl-acid formation and incubated using anaerobic digester sludge as inoculum (Figure 2). Tryptophan, tyrosine, and phenylalanine, as well as the complex proteinaceous precursor meat extract and casein were applied in two and three different concentrations, respectively. Controls were prepared without additional substrate supplementation. Different substrate concentrations aimed at the simulation of different stages of overload. Flasks were incubated at 37°C (mesophilic) for 4 weeks.

**Figure 3:**
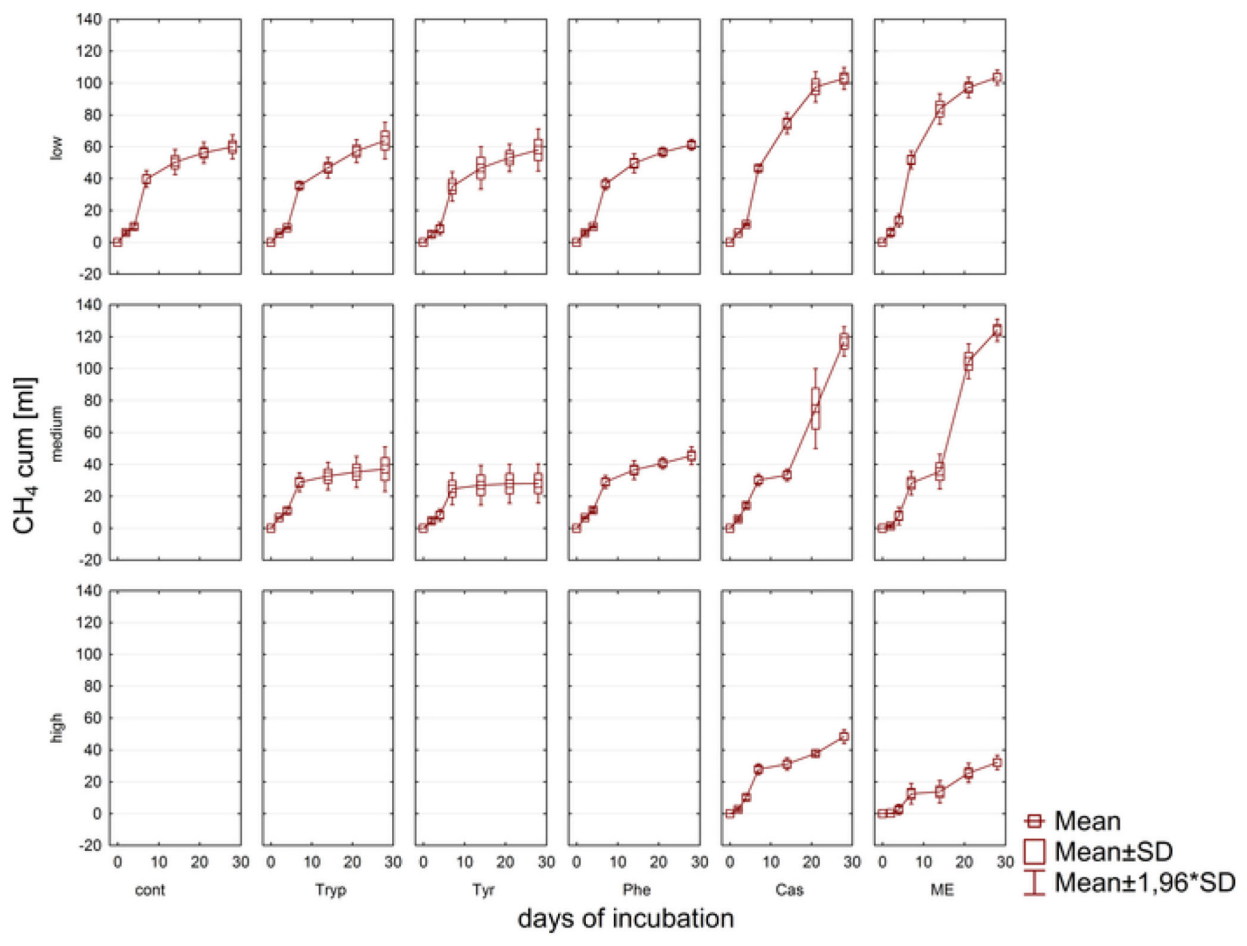
Methane production. Cumulative methane production during 28 days of mesophilic incubation from reactors reflecting different overload conditions (low, medium, high). Cont: control; Tryp: tryptophan; Tyr: tyrosine; Phe: phenylalanine; ME: meat extract; Cas: casein. This is a modified figure originating from an earlier study^8^. Please click here to view a larger version of this figure.

**Figure 4:**
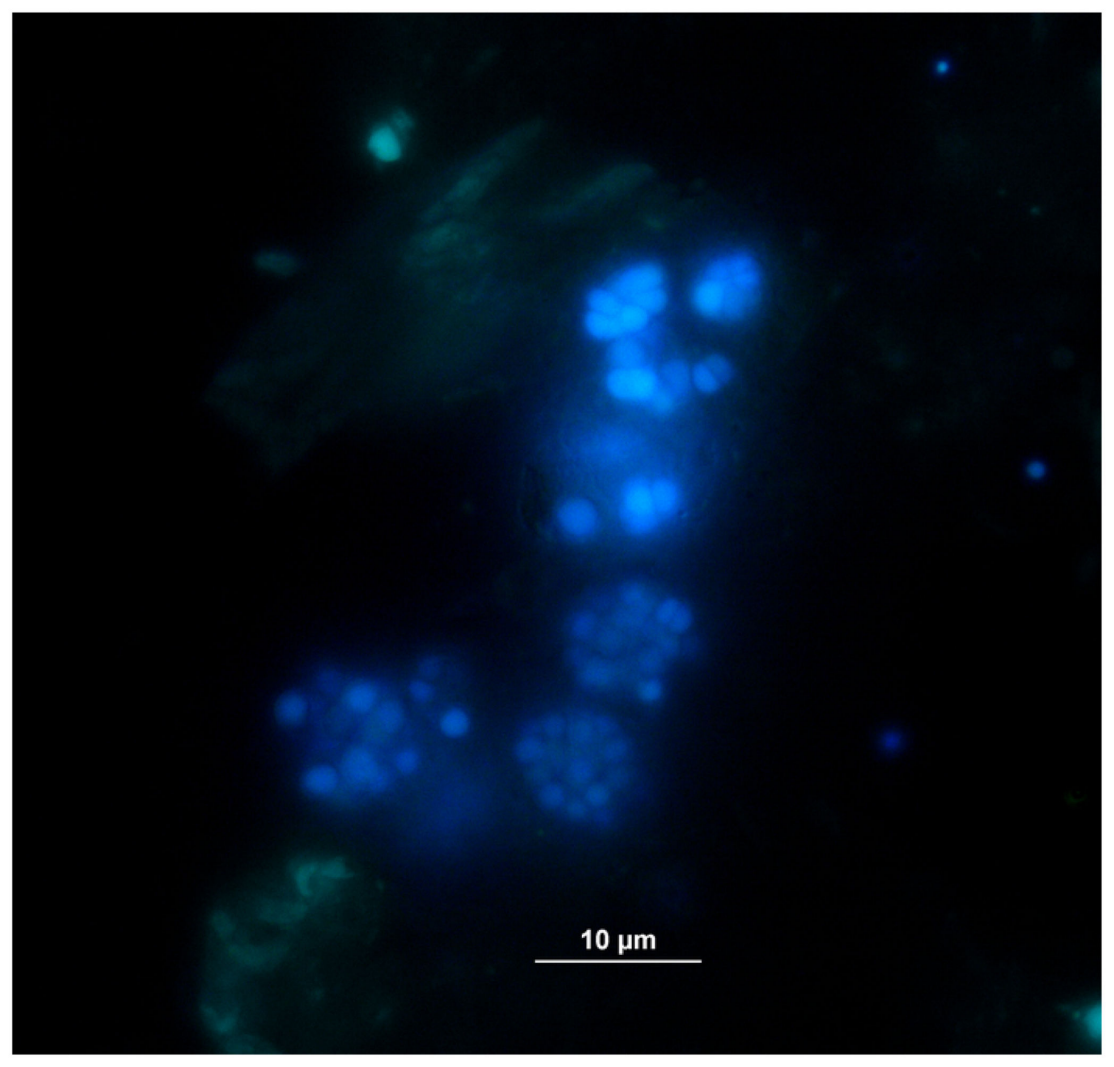
Fluorescing methanogens. Methanogens emit a blueish light when being excited with UV light. Here, methanogens are attached to plant particles (light green). Samples were taken from a batch reactor, diluted for microscopy, and immediately analyzed. Please click here to view a larger version of this figure.

Biogas production and composition (H_2_, CH_4_, CO_2_) was monitored regularly via gas chromatography (GC TCD)^10^ and evaluation of headspace pressure. Figure 3 demonstrates differences in the cumulative methane production derived from digestion of the applied substrates in varied concentrations during 4 weeks of anaerobic incubation. Besides, methanogens were visualized by irradiating the coenzyme F_420_, an electron carrier in methanogenesis, exhibiting a blue-green fluorescence with an absorption maximum at 420 nm (Figure 4).

**Figure 5:**
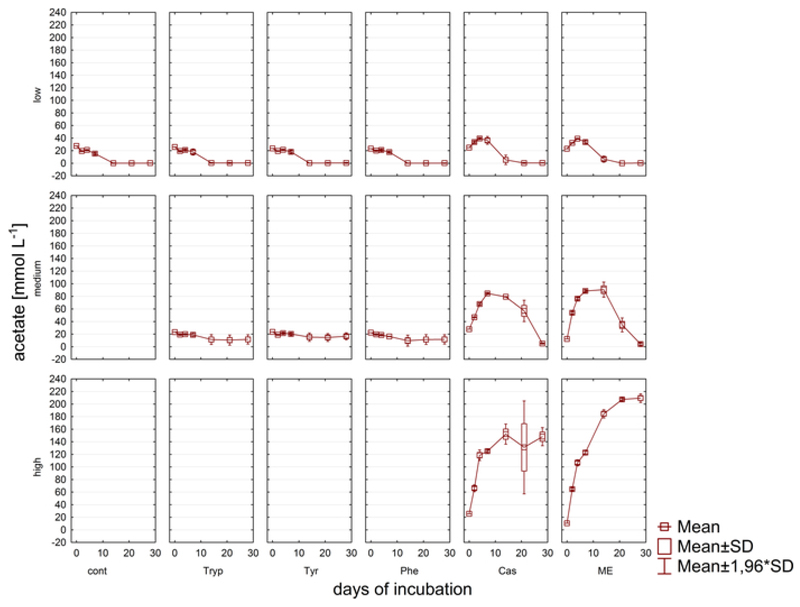
Acetate concentration. Acetate concentration during 28 days of mesophilic incubation in reactors reflecting different overload conditions (low, medium, high). Cont: control; Tryp: tryptophan; Tyr: tyrosine; Phe: phenylalanine; ME: meat extract; Cas: casein. This is a modified figure originating from an earlier study^8^. Please click here to view a larger version of this figure.

**Figure 6:**
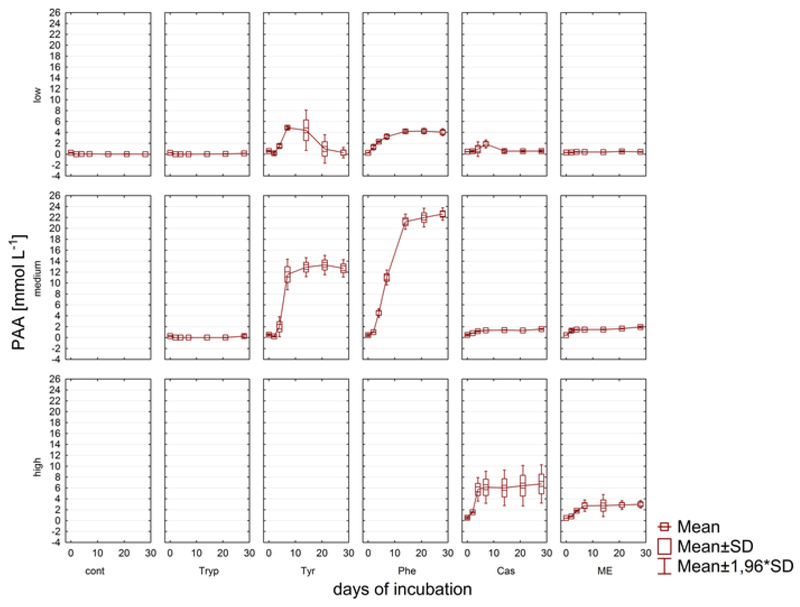
Phenylacetate concentration. Phenylacetate concentration during 28 days of mesophilic incubation in reactors reflecting different overload conditions (low, medium, high). Cont: control; Tryp: tryptophan; Tyr: tyrosine; Phe: phenylalanine; ME: meat extract; Cas: casein. This is a modified figure originating from an earlier study^8^. Please click here to view a larger version of this figure.

Concurrent to gas analysis, samples for VFA and phenyl acid concentration measurements via HPLC^11^ were withdrawn and stored frozen until further processing. Figure 5 shows the effect of different stages of overload as reflected by an accumulation in highly overloaded samples exemplarily depicted for acetate. Figure 6 depicts the dynamics of phenyl acetate concentrations during the incubation period.

## Discussion

**Figure 7:**
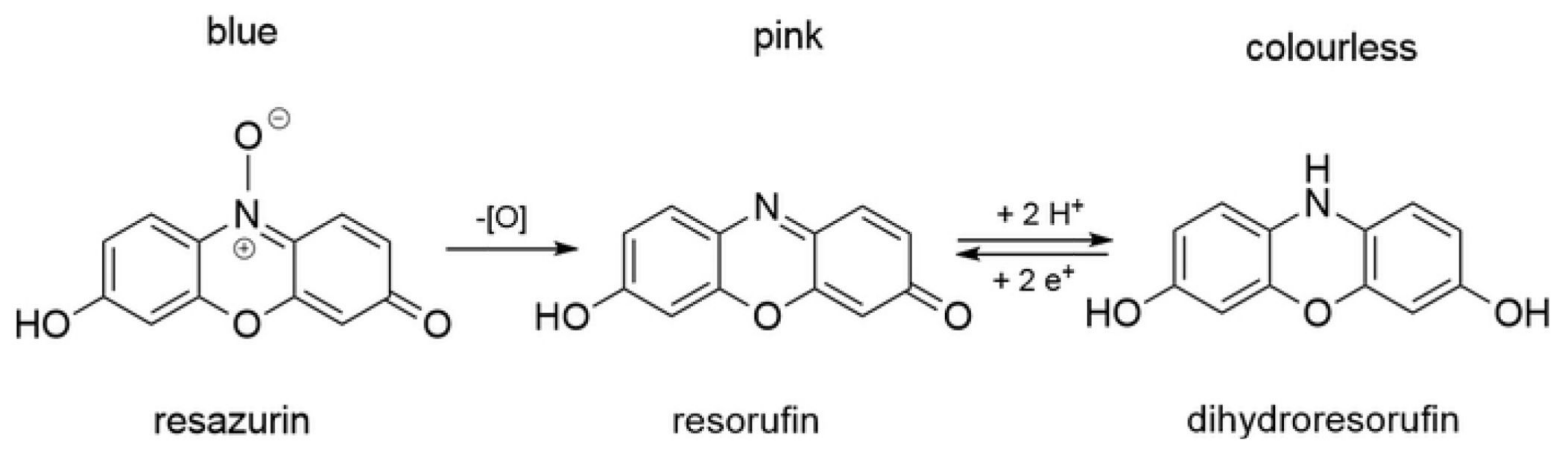
Resazurin reaction. Blue colored resazurin undergoes an irreversible reduction to resorufin (pink) and a further reversible reduction to the colourless dihydroresorufin according to Uzarski et al.^12^. Please click here to view a larger version of this figure.

The most important and critical step in culturing anaerobic microorganisms is to ensure oxygen-free conditions in cultivation media and flasks’ headspace. An indicator like resazurin can be used to indirectly check the correct anaerobic filling of the flasks. Resazurin is a commonly used redox dye as it is inexpensive, non-toxic, and already effective in low doses and short incubation times ^12^. When incorporated to media, the blue colored resazurin first undergoes an irreversible reduction step to resorufin, which is pink at neutral pH values. This first reaction can occur when the media are heated 13. Subsequently, resorufin is reduced to colorless dihydroresorufin in a reversible secondary reaction (Figure 7)^12^. The resorufin/dihydroresorufin redox system becomes completely colorless at a standard oxidation-reduction potential of about Eh = -110 mV and turns pink above a redox potential of -51 mV ^13^.

In order to further reduce the redox potential, for example, to facilitate the growth of methanogenic microorganisms known to require less than -200 mV^14^, a Na_2_S solution can be added. Alternatively, cysteine-HCl, sodium-thioglycolate, or sodium dithionite are commonly used. However, which reducing agent is appropriate to use depends on the respective experimental setup and might require special attention. For instance, sodium thioglycolate needs temperature activation (e.g., by autoclaving).

A well-balanced microbial consortium, comprised of various genera of Bacteria and Archaea, and an efficiently working anaerobic degradation cascade can further be evaluated by determining the headspace gas composition in the culture flasks via gas chromatography. When handling compounds like phenyl acids derived from different precursors, the assessment of the headspace is a fast way to check the methanogenesis process^8^. A headspace CH4 concentration of approx. 50-60% in the controls at the end of the incubation period indicates a successful utilization of the applied nutrients and thus a mineralization of organic material under anaerobic conditions. The theoretical methane production and expectable methane concentrations during the digestion process can be determined *ex ante* according to the Buswell-Boyle equation after elementary analysis of the substrate or by estimating the content of carbohydrates, proteins, and fats in the substrate. According to VDI 4630 ^15^, carbohydrates can lead to a theoretical biogas production of 750 L kg^-1^ VSS (50% CH_4_ and 50% CO_2_), proteins to 800 L kg^-1^ VSS (72% CH_4_ and 28% CO_2_), and fats to 1,390 L kg^-1^ VSS (60% CH_4_ and 40% CO_2_).

Moreover, formation and possible subsequent degradation of VFAs and phenyl acids were monitored. The degradation process can be evaluated by analyzing the VFA concentrations (e.g., acetate, propionate) at different time points. Accumulation of short-chain fatty acids like acetate and/or propionate can point to disturbances in the methanogenic community composition and to an overall reactor overload. However, a well-balanced microbial degradation cascade can even cope with very high VFA and acetate concentrations^9^. Besides, the acetate / propionate ratio might further provide information on the overall reactor condition^16^. However, there are many parameters suitable for process monitoring that have to be selected according to the proposed experimental hypotheses. In the present example, target variables were phenyl acid concentrations (Figure 6).

## Figures and Tables

**Table 1:** Vitamin solution.

Cyanocobalamin	0.050 g
4-aminobenzoic acid	0.050 g
D-biotin	0.010 g
Nicotinic acid	0.100 g
Pyridoxine	0.250 g
D-pantothenic acid	0.025 g
Thiaminium chloride HCl	0.18 g
Distilled water	1000 mL

**Table 2:** Trace element solution.

25% (w/v) HCl	10.0 mL
FeCl_2_ x 4 H_2_O	1.50 g
ZnCl_2_	0.070 g
MnCl_2_ x 4 H_2_O	0.100 g
H_3_BO_3_	0.006 g
CoCl_2_ x 6 H_2_O	0.190 g
CuCl_2_ x 2 H_2_O	0.002 g
NiCl_2_ x 6 H_2_O	0.024 g
Na_2_MoO_4_ x 2 H_2_O	0.036 g
Distilled water	990.0 mL
Preparation recommendation	Add HCl and dissolve FeCl_2_, add 100 mL distilled water, dissolve the other ingredients, and make up to 1000 mL.

**Table 3:** Minimal salt medium.

NaCl	1.0 g
MgCl_2_ x 6 H_2_O	0.4 g
KH_2_PO_4_	0.2 g
KCl	0.5 g
CaCl_2_ x 2 H_2_O	0.15 g
L-cysteine	0.5 g
Yeast extract	1.0 g
Resazurin solution	1 mL
Vitamin solution	1 mL
Trace element solution	1 mL
Distilled water	1000 mL
pH	7.2

**Table 4:** Description of variables in Equation 1 - 3.

Variable	Unit	Description
t_Y_	[d]	Timepoint of measurement
t_X_	[d]	Timepoint of preceding measurement
p_M_	[mbar]	Measured overpressure at t_Y_
p_A_	[mbar]	Ambient pressure at t_Y_
p_AX_	[mbar]	Ambient pressure at t_X_
p_S_	[mbar]	Standard pressure, 1013,25 mbar acc. DIN 1343
T_I_	[K]	Incubation temperature
T_S_	[K]	Standard temperature, 273,15 K (corresponds to 0°C) acc. DIN 1343
V_H_	[ml]	Headspace volume at t_Y_
V_HX_	[ml]	Headspace volume at t_X_
CH_4%_	[vol%]	Methane concentration according to GC-measurement at t_Y_
CH_4%X_	[vol%]	Methane concentration according to GC-measurement at t_X_
V_CH4T_	[Nml]	Total methane amount in the serum bottle at t_Y_
V_CH4R_	[Nml]	Residual methane amount in the headspace at t_X_
V_CH4N_	[Nml]	Newly produced methane from t_X_ to t_Y_
